# Multimodal diaphragmatic ultrasound indicators in healthy adults: reliability and consistency observation by ultrasound physician and critical care physician

**DOI:** 10.3389/fmed.2025.1542979

**Published:** 2025-03-31

**Authors:** Haotian Zhao, Ling Long, Yi Liu, Yaru Yan, Xiaona Wang, Heling Zhao, Li Li

**Affiliations:** ^1^Department of Ultrasound, Hebei General Hospital, Shijiazhuang, China; ^2^Department of Intensive Care Unit, Hebei General Hospital, Shijiazhuang, China; ^3^Department of Ultrasound, Shijiazhuang People’ Hospital, Shijiazhuang, China

**Keywords:** diaphragmatic function, ultrasound examination, shear wave elastography, normal values, healthy adults

## Abstract

**Background:**

This study aimed to establish normal reference values for multimodal diaphragmatic ultrasound indices in healthy adult volunteers and evaluate intra-and inter-observer consistency between ultrasound physicians and critical care physicians.

**Methods:**

An ultrasound physician (US physician) used techniques such as B-mode, M-mode, Tissue Doppler Imaging (TDI), and shear wave elastography (SWE) to measure diaphragmatic parameters in 46 healthy adults during quiet and deep breathing. A critical care physician (CC physician) trained in diaphragmatic ultrasound repeated these measurements. Consistency was analyzed in intra-researcher and inter-researchers of various diaphragmatic ultrasound indicators.

**Results:**

Diaphragm thickness at the end of expiration, end of inspiration, and end of deep inspiration (DT-ee, DT-ei, and DT-edi) in both B-mode and M-mode method of males were higher than that of females, Diaphragm shear modulus-edi of males is higher than that of females (*P*<0.05). Between different diaphragmatic ultrasound indicators, the study showed a moderate consistency of peak contraction velocity (PCV) and peak relaxation velocity (PRV) in intra-researcher of CC physician and inter-researchers between US physician and CC physician, (ICC = 0.678 and 0.704, *P* < 0.001). For other multiple diaphragm ultrasound indicators, our study showed an excellent consistency in both intra-researcher and inter-researchers (ICC = 0.824–0.994, *P* < 0.001). For DT measurement by B-mode and M-mode, it showed an excellent consistency in both intra-researcher, intra-researcher of US physician, intra-researcher of CC physician and total cases (ICC = 0.919–0.960, *P* < 0.001). Correlation analysis showed a moderate positive correlation between diaphragm displacement during quiet breathing (DD-qb) and pleural sliding displacement (PSD) in US physician (*r* = 0.568), CC physician (*r* = 0.470), and total cases (*r* = 0.511), with significant differences (*P* < 0.05).

**Conclusion:**

Ultrasound-based assessment of diaphragmatic function is a reliable method. This study provides normal reference values and highlights the high observer reproducibility among experienced ultrasound and critical care physicians.

## Introduction

The diaphragm, the primary muscle responsible for inspiration, contributes approximately 80% of the respiratory workload (1, 2). Ultrasound plays a significant role in assessing diaphragmatic structure and function in various conditions, such as chronic obstructive pulmonary disease (COPD), ventilator-induced diaphragmatic atrophy, and post-stroke hemiplegia (3). In intensive care unit (ICU) patients, factors like mechanical ventilation, protein imbalance, and sepsis-induced inflammation can lead to muscle degradation (4, 5), with the diaphragm being particularly susceptible due to its thin structure and rapid atrophy compared to skeletal muscles. Diaphragmatic disuse atrophy may occur shortly after receiving mechanical ventilation in ICU hospitalization, which may lead to subsequent weaning failure (4).

Diaphragmatic ultrasound commonly employs two key indices: diaphragm displacement (DD), which reflects lung ventilation through diaphragmatic motion (6), and diaphragm thickening fraction (DTF), indicating changes in diaphragm thickness during inspiration (7). Advances in ultrasound technology have introduced newer parameters, such as TDI for contraction velocity during systole and diastole and SWE for measuring diaphragmatic stiffness (8, 9). However, studies focusing on normal values for these indices are limited. Establishing normal values is crucial for assessing diaphragmatic function. Although several studies have determined the normal values of DD or DTF measured in healthy volunteers in supine position or in sitting position (10–13). However, Multimodal ultrasound methods should be included.

A novel diaphragmatic ultrasound parameter, pleural sliding displacement (PSD), measures pleural sliding at the lung-liver boundary, representing diaphragmatic mobility. It may serve as an alternative indicator in patients with obesity, high intra-abdominal pressure, or post-abdominal surgery.

In practice, there are two ways to assess diaphragm function in different regions or hospitals. One is for CC physicians to learn ultrasound technology and apply it in the ICU. Another method is to provide diaphragmatic ultrasound examination by a US physician, including patients in ICU or general wards, and healthy individuals. These two methods complement each other. This study aims to determine normal reference values for various diaphragmatic ultrasound indices and analyze inter-and intra-observer consistency across different parameters and sexes.

## Materials and methods

### Participants

This prospective observational study was conducted at a tertiary hospital from April 30 to August 20, 2024, including 46 healthy adults (23 males, 23 females). Inclusion criteria: (1) Age ≥ 18 years old and ≤ 45 years old; (2) Respiratory function is normal, and all patients have undergone lung ultrasound or pulmonary CT examination without any pulmonary diseases. Exclusion criteria included acute or chronic cardiac or pulmonary disease, cardiomyopathy, congenital heart disease, pulmonary interstitial fibrosis, COPD, neuromuscular disorders, recent chest or abdominal surgery, pregnant, or invasive mechanical ventilation within 3 months, or other factors deemed unsuitable for participation in the study.

This study aimed to establish normal reference values for multimodal diaphragmatic ultrasound indices in healthy adult volunteers and evaluate intra-and inter-observer consistency between ultrasound physicians and critical care physicians. The calculation of sample size was using bivariate statistical correlation analysis with a power of 80% and *α* error of 0.05 referred to previous studies evaluating diaphragm ultrasound measurement (14). A total sample size of 46 participants was obtained as the required sample size.

### Diaphragmatic ultrasound examination

This study utilized a color Doppler ultrasound system (EPIQ ELITE, Philips, Netherlands) equipped with a phased array probe (1–5 MHz) and a high-frequency linear array probe (4–18 MHz). The primary researcher, an experienced US physician, with experience in performing diaphragmatic ultrasound over 1,500 times (patients mainly from ICU, department of respiratory, department of emergency, department of rehabilitation, etc.). The secondary investigator, a CC physician, with experience in performing diaphragmatic ultrasound over 500 times (patients mainly from ICU). Ultrasound parameters were assessed under different respiratory patterns. All participants underwent breathing training before diaphragmatic ultrasound to ensure proper execution of quiet and deep breathing techniques. Participants fasted for at least 6 h before the examination. During the procedure, they maintained steady quiet breathing unless instructed otherwise. For deep breathing maneuvers, participants began inhaling at the end of a quiet exhalation and continued until maximal inspiratory effort was reached.

Before initiating the study, the two researchers discussed the selection of ultrasound probe placement sites on five healthy participants. After reaching a consensus on the operation method and site selection, the study commenced. The US physician conducted the initial measurements, obtaining at least three stable waveforms before data collection. Ultrasound parameters were measured over at least three respiratory cycles, and the average values were recorded. Subsequently, the CC physician performed the same measurements. After a 15-min, both researchers independently repeated the above measurements. An independent physician, blinded to the results, recorded all data.

### Assessment of DD and diaphragm thickness

**Figure 1:**
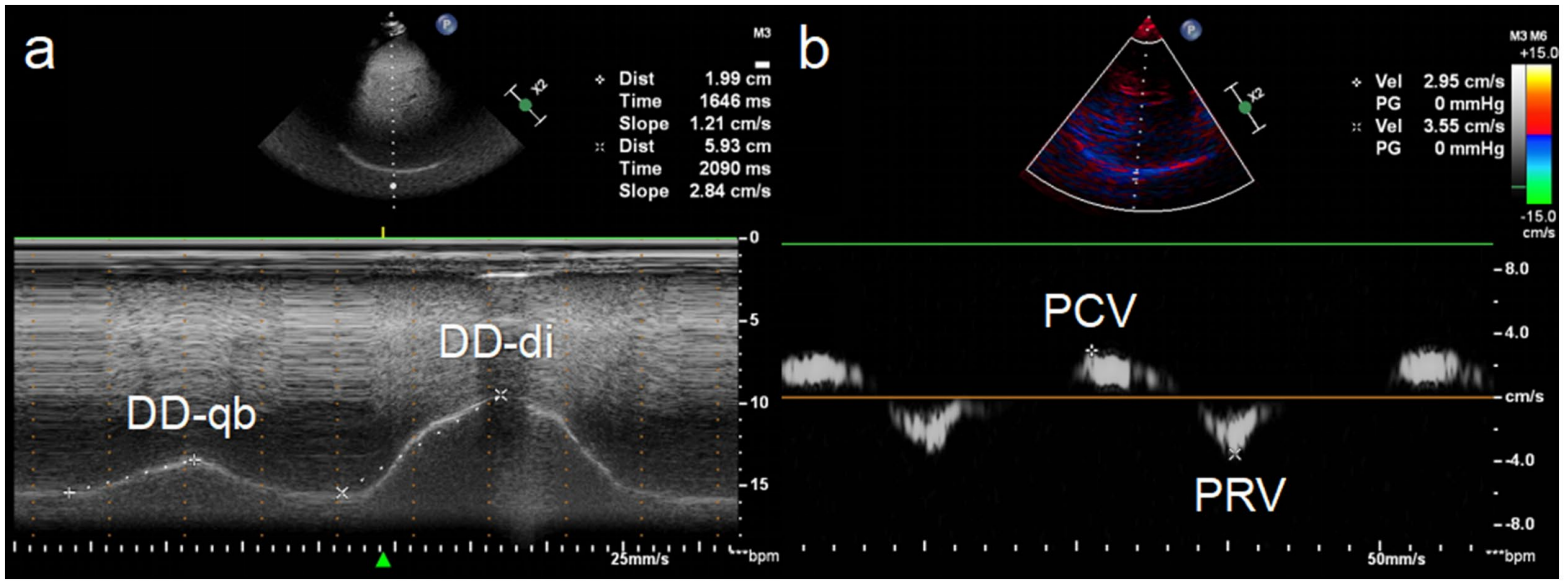
Ultrasonic measurement of diaphragm displacement (DD) and peak velocity. **(a)** The measured value of diaphragm displacement during quiet breathing (DD-qb) was 19.9 mm and during deep inspiration (DD-di) was 59.3 mm. **(b)** The measured value of peak contraction velocity (PCV) during inspiration was 2.95 cm/s and peak relaxation velocity (PRV) during expiration was 3.55 cm/s.

Measurements were performed using a phased array 1–5 MHz probe placed between the right midclavicular and anterior axillary lines at the subcostal margin, oriented cranially. DD during quiet breathing (DD-qb) was observed, and maximum DD during deep inspiration (DD-di) was measured as the difference between end expiration and end deep inspiration positions (Figure 1a).

Tissue Doppler imaging (TDI) was employed to assess diaphragmatic contraction by positioning the sample box at the diaphragm apex, the same site used for DD measurement. The sample was adjusted by the operator in order to include the diaphragm for TDI velocities recording. Peak contraction velocity (PCV) during inspiration and peak relaxation velocity (PRV) during expiration were recorded (Figure 1b).

**Figure 2:**
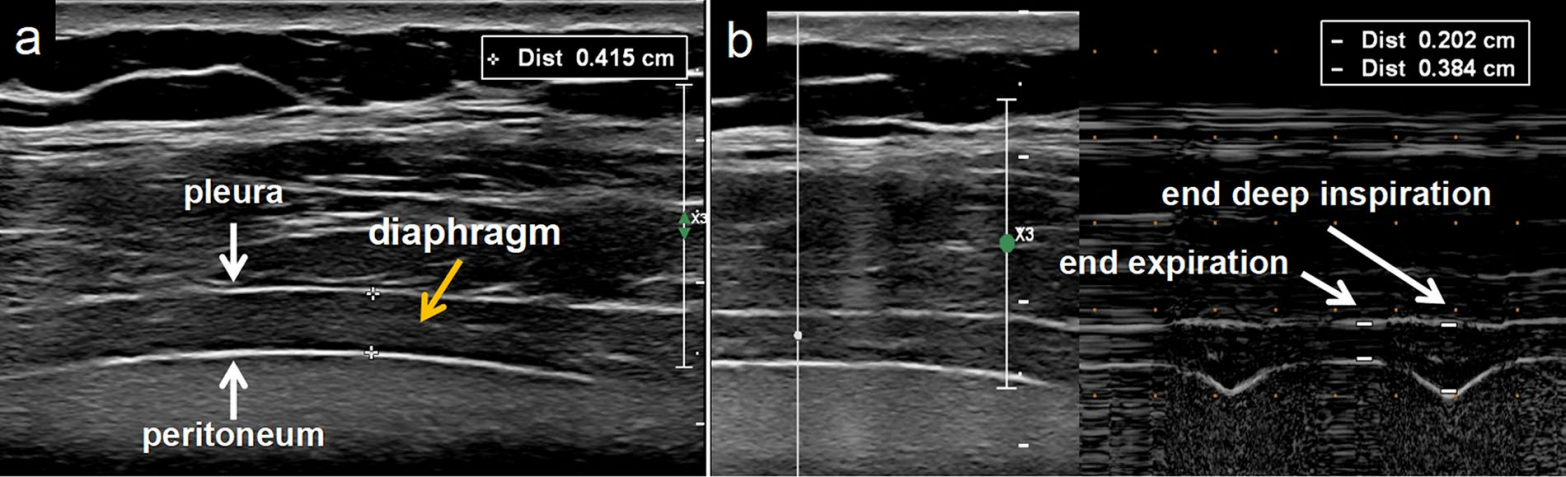
Diaphragm thickness measurement in B-mode and M-mode. **(a)** The diaphragm (yellow arrow) is the muscle tissue between the pleura (downwards white arrow) and peritoneum (upwards white arrow) measured using B-mode. **(b)** Diaphragm thickness measured by M-mode.

High-frequency linear array 4–18 MHz probe was placed along the right midaxillary line in the 8th to 10th intercostal spaces to obtain DT. DT was measured at the pleural-peritoneal interface at the end of expiration (DT-ee), end of inspiration (DT-ei), and end of deep inspiration (DT-edi). The measurement of DT should be conducted using B-mode and M-mode, respectively, (Figure 2).

### Assessment of pleural sliding displacement

**Figure 3:**
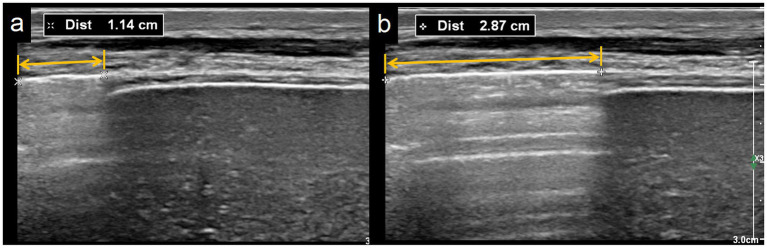
The distance between lung-liver boundary and the left edge of the sound beam on the pleural line**. (a)** At the end expiration, the measured value is 11.4 mm. **(b)** At the end inspiration, the measured value is 28.7 mm, and the calculated of pleural sliding displacement (PSD) is 28.7–11.4 = 17.3 mm.

Using a linear array 4–18 MHz probe placed along the right midaxillary line, the liver and lung interface was visualized. The liver appeared as a homogenous structure, while the lung displayed a pleural line with posterior A-lines (artifacts). The pleura moved toward the liver during inspiration and away during expiration, a phenomenon termed the “curtain sign” (Figure 3). The distance between the lung-liver interface and the left boundary of the acoustic window was measured, and the difference was PSD, represented the movement of the lung’s lower boundary during the respiratory cycle.

### Shear wave elastography mode

**Figure 4:**
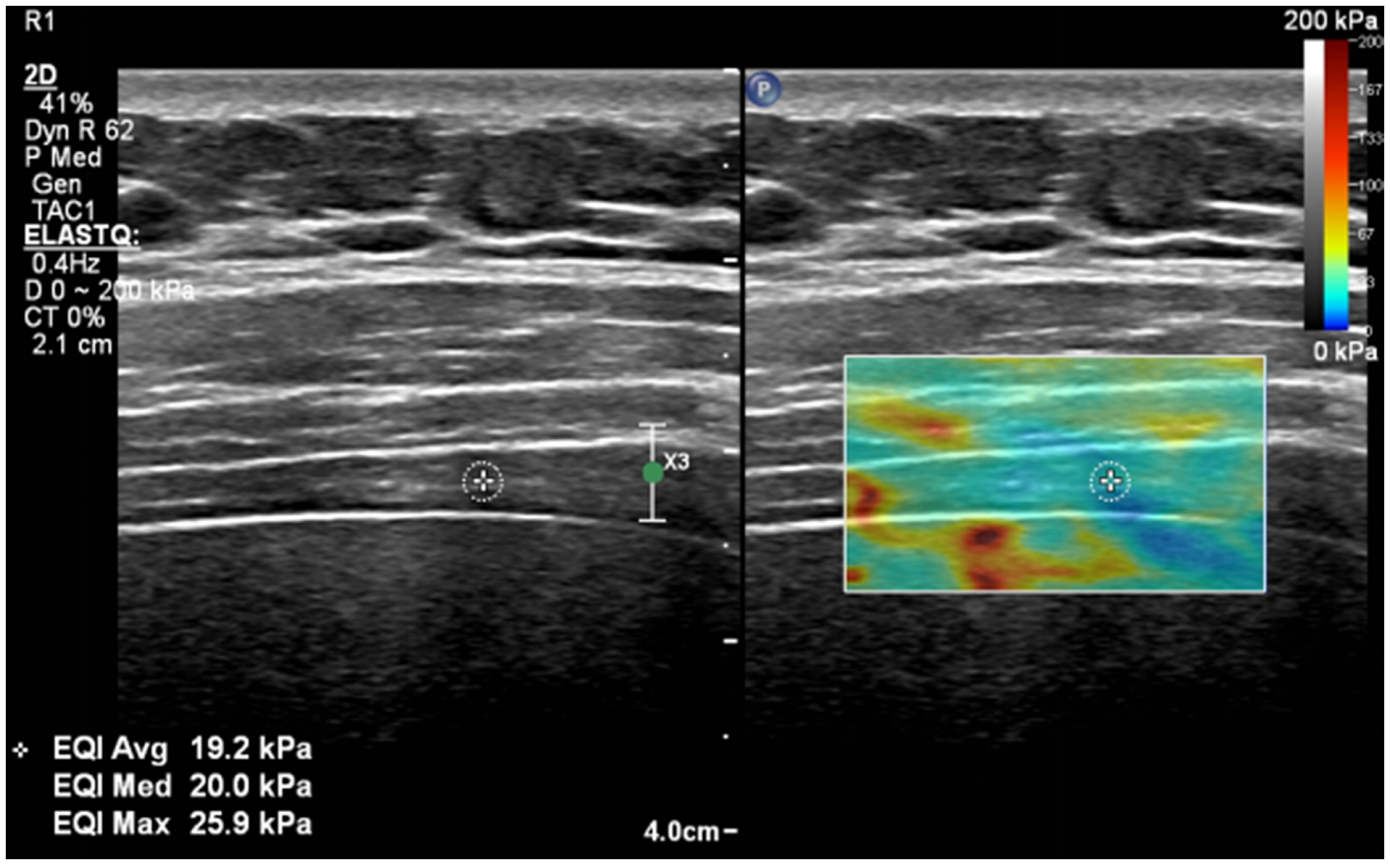
Shear wave elastography technique for evaluating diaphragm.

Using SWE mode of the ultrasound scanner, and the measurement position is still the same as the diaphragm thickness. The region of interest should be placed at the position to fully cover the diaphragm, and avoid obvious muscle fiber. The participant was required to stop breathing at the end-expiration of quiet breathing and maintain it for about 5 s, and measure the diaphragm shear modulus at the end expiration (diaphragm shear modulus-ee). Subsequently, the participant was required to perform maximum inspiration as previously trained, and measured the diaphragm shear module at the end of deep inspiration (diaphragm shear modulus-edi) (Figure 4). The measurements were repeated a minimum of three and a maximum of five times.

### Clinical and biological data

Age, gender, height, weight, body mass index (BMI), respiratory rate, heart rate, systolic and diastolic blood pressure were recorded.

### Statistical analysis

The Statistical Package of Social Sciences (SPSS), version 21.0 (IBM; Armonk, NY, United States) software package was used for data analysis. Continuous variables were expressed as a mean ± standard deviation (SD) or median (IQR) according to the data distribution and analyzed by Mann–Whitney *U* tests or *t-*tests. Categorical variables were expressed as frequencies (percentages). The repeatability of measurements of diaphragm ultrasound indicators of intra-researcher (each researcher), inter-researchers (between the two researchers) were analyzed. Intraclass Correlation Coefficient (ICC) were calculated and classified as poor (ICC < 0.40), weak (ICC = 0.40–0.59), good (ICC = 0.60–0.74), and excellent (ICC = 0.75–1.00). Correlation analysis was conducted using Spearman’s method, and classified as weak (*r* < 0.40), moderate (*r* = 0.40–0.59), good (*r* = 0.60–0.79), and excellent (*r* = 0.80–1.00). *p* < 0.05 was considered to concede statistical significance.

## Results

### Populations

A total of 52 participants underwent diaphragmatic ultrasound. Two were excluded due to suboptimal imaging, three failed to perform the required breathing techniques, and 1 did not adhere to the fasting protocol. Ultimately, 46 participants (23 males, 23 females) were included. Their ages ranged from 19 to 45 years, and BMI ranged from 17.65 to 28.08 kg/m^2^. All participants had normal respiratory and heart rates, a participant with grade 1 hypertension, but no cardiovascular or pulmonary disease, while all others had normal blood pressure. The basic characteristics of all participants are presented in Table 1.

**Table 1:** Characteristics of the study participants.

Characteristics	Male (*n* = 23)	Female (*n* = 23)	Statistical value	*P*-value
Age (years)	31.65 ± 7.63	32.91 ± 5.09	*t* = −0.659	0.513
Height (cm)	1.74 (1.72, 1.77)	1.61 (1.60, 1.67)	*Z* = −5.462	<0.001
Weight (kg)	76.37 ± 8.91	59.34 ± 6.68	*t* = 7.334	<0.001
BMI (kg/m^2^)	25.02 ± 2.39	22.47 ± 2.67	*t* = 3.414	0.001
Respiratory rate (times per minute)	15.04 ± 2.62	15.52 ± 1.73	*t* = −0.731	0.469
Heart rate (times per minute)	76.00 ± 10.51	70.61 ± 7.07	*t* = 2.042	0.047
Systolic blood pressure (mmHg)	121.00 (117.00, 124.00)	117.00 (112.00, 126.00)	*Z* = −1.653	0.098
Diastolic blood pressure (mmHg)	70.30 ± 7.31	66.96 ± 7.00	*t* = 1.586	0.120

BMI, body mass index.

### Diaphragm ultrasound study

Table 2 reports the normal values and reference ranges of various indicators of diaphragmatic ultrasound. Compared to females, males have higher levels of DT-ee, DT-ei, DT-edi in both B-mode and M-mode methods of measurement (*P* < 0.05). The diaphragm shear modulus-edi of males is higher than that of females (*P* < 0.05) There were no significant statistical differences among the other indicators (*P* > 0.05).

**Table 2:** Diaphragmatic ultrasound measurement indicators [mean ± SD or median (IQR)].

	Total (*n* = 46)	Male (*n* = 23)	Female (*n* = 23)	Statistical value	*P*-value
B-mode					
DD-qb (mm)	15.51 ± 3.45	16.25 ± 4.05	14.77 ± 2.61	*t* = 1.480	*p* = 0.146
DD-di (mm)	55.95 ± 10.47	57.04 ± 11.34	54.86 ± 9.65	*t* = 0.701	*p* = 0.487
PSD (mm)	17.20 ± 4.96	17.46 ± 5.50	16.94 ± 4.47	*t* = 0.350	*p* = 0.728
DT-ee (mm)	1.75 (1.48, 1.94)	1.85 (1.75, 2.33)	1.50 (1.24, 1.73)	*Z* = −4.617	*P*<0.001
DT-ei (mm)	2.20 ± 0.54	2.54 ± 0.43	1.85 ± 0.40	*t* = 5.677	*P*<0.001
DT-edi (mm)	3.68 ± 0.95	4.27 ± 0.77	3.09 ± 0.73	*t* = 5.331	*P*<0.001
M-mode					
DT-ee (mm)	1.69 ± 0.39	1.94 ± 0.31	1.44 ± 0.29	*t* = 5.735	*P*<0.001
DT-ei (mm)	2.09 (1.71, 2.72)	2.50 (2.29, 2.97)	1.72 (1.53, 1.97)	*Z* = −4.625	*P*<0.001
DT-edi (mm)	3.71 (3.12, 4.22)	4.21 (3.75, 4.73)	3.20 (2.49, 3.71)	*Z* = −4.614	*P*<0.001
TDI-mode					
PCV (cm/s)	2.32 ± 0.27	2.34 ± 0.30	2.30 ± 0.25	*t* = 0.449	*p* = 0.656
PRV (cm/s)	2.09 ± 0.36	2.11 ± 0.39	2.06 ± 0.34	*t* = 0.450	*p* = 0.655
SWE-mode					
Diaphragm shear modulus-ee (kPa)	15.37 ± 2.64	15.83 ± 2.01	14.92 ± 3.13	*t* = 1.172	*p* = 0.247
Diaphragm shear modulus-edi (kPa)	20.35 (19.10, 23.53)	22.40 (19.90, 25.00)	19.90 (17.50, 22.20)	*Z* = −2.220	*p* = 0.026

DD-qb, diaphragm displacement during quiet breathing; DD-di, diaphragm displacement during deep inspiration; PSD, pleural sliding displacement; DT-ee, diaphragm thickness at the end of expiration; DT-ei, diaphragm thickness at the end of inspiration; DT-edi, diaphragm thickness at the end of deep inspiration; PCV, peak contraction velocity; PRV, peak relaxation velocity; SWE, shear wave elastography.

### Repeatability test in intra-and inter-researcher

The study showed a moderate consistency of PCV and PRV in intra-researcher of CC physician (ICC = 0.687 and 0.678, *P*<0.001), a moderate consistency of PCV and PRV in inter-researchers between US physician and CC physician (ICC = 0.702 and 0.704, *P* < 0.001) and an excellent consistency of PCV and PRV in intra-researcher of US physician (ICC = 0.884 and 0.863, *P* < 0.001). For other multiple diaphragm ultrasound indicators, our study showed an excellent consistency in both intra-researcher and inter-researchers (ICC = 0.824–0.994, *P* < 0.001) (Table 3).

**Table 3:** Repeatability test in intra-researcher and inter-researchers (ICC).

	DD-qb	DD-di	PSD	PCV	PRV	DT-ee(B)	DT-ee(M)	DT-ei(B)	DT-ei(M)	DT-edi(B)	DT-edi(M)	SWE-ee	SWE-edi
Intra-US physician, *n* = 46													
ICC	0.972 (0.950 ~ 0.985)	0.955 (0.919 ~ 0.975)	0.932 (0.880 ~ 0.962)	0.884 (0.800 ~ 0.934)	0.863 (0.766 ~ 0.922)	0.994 (0.988 ~ 0.996)	0.980 (0.964 ~ 0.989)	0.994 (0.989 ~ 0.996)	0.974 (0.953 ~ 0.985)	0.988 (0.979 ~ 0.993)	0.969 (0.945 ~ 0.983)	0.947 (0.906 ~ 0.970)	0.908 (0.839 ~ 0.948)
*P*	<0.001	<0.001	<0.001	<0.001	<0.001	<0.001	<0.001	<0.001	<0.001	<0.001	<0.001	<0.001	<0.001
Intra-CC physician, n = 46													
ICC	0.964 (0.935 ~ 0.980)	0.956 (0.921 ~ 0.975)	0.886 (0.788 ~ 0.938)	0.687 (0.497 ~ 0.814)	0.678 (0.483 ~ 0.808)	0.985 (0.974 ~ 0.992)	0.941 (0.896 ~ 0.967)	0.982 (0.966 ~ 0.990)	0.945 (0.904 ~ 0.969)	0.982 (0.967 ~ 0.990)	0.968 (0.944 ~ 0.982)	0.876 (0.786 ~ 0.929)	0.883 (0.798 ~ 0.933)
*P*	<0.001	<0.001	<0.001	<0.001	<0.001	<0.001	<0.001	<0.001	<0.001	<0.001	<0.001	<0.001	<0.001
Inter-US physician and CC physician, n = 92													
ICC	0.927 (0.890 ~ 0.951)	0.911 (0.868 ~ 0.940)	0.840 (0.768 ~ 0.891)	0.702 (0.582 ~ 0.792)	0.704 (0.583 ~ 0.794)	0.977 (0.966 ~ 0.985)	0.935 (0.903 ~ 0.956)	0.976 (0.964 ~ 0.984)	0.938 (0.907 ~ 0.958)	0.967 (0.950 ~ 0.978)	0.942 (0.914 ~ 0.961)	0.824 (0.745 ~ 0.880)	0.874 (0.815 ~ 0.915)
*P*	<0.001	<0.001	<0.001	<0.001	<0.001	<0.001	<0.001	<0.001	<0.001	<0.001	<0.001	<0.001	<0.001

DD-qb, diaphragm displacement during quiet breathing; DD-di, diaphragm displacement during deep inspiration; PSD, pleural sliding displacement; DT-ee, diaphragm thickness at the end of expiration; DT-ei, diaphragm thickness at the end of inspiration; DT-edi, diaphragm thickness at the end of deep inspiration; PCV, peak contraction velocity; PRV, peak relaxation velocity; SWE, shear wave elastography; ICC, Intraclass Correlation Coefficient; US physician, Ultrasound physician; CC physician, Critical Care physician.

### Repeatability test between B-mode and M-mode measurement of diaphragm thickness

We also measured the diaphragm thickness at three breathing phases, respectively, using the two methods of B-mode and M-mode. It showed an excellent consistency in both intra-researcher, intra-researcher of US physician, intra-researcher of CC physician and total cases (ICC = 0.919–0.960, *P*<0.001). There is no statistical difference between the two physicians (*P* > 0.05) (Table 4).

**Table 4:** Repeatability test between B-mode and M-mode measurement of diaphragm thickness (ICC).

	DT-ee (B and M mode)		DT-ei (B and M mode)		DT-edi (B and M mode)	
	ICC	*P*	ICC	*P*	ICC	*P*
Intra-US physician, *n* = 92	0.960 (0.933 ~ 0.975)	<0.001	0.938 (0.908 ~ 0.959)	<0.001	0.950 (0.925 ~ 0.967)	<0.001
Intra-CC physician, *n* = 92	0.919 (0.880 ~ 0.945)	<0.001	0.939 (0.910 ~ 0.959)	<0.001	0.940 (0.904 ~ 0.961)	<0.001
Total, *n* = 184	0.939 (0.917 ~ 0.955)	<0.001	0.939 (0.919 ~ 0.954)	<0.001	0.944 (0.923 ~ 0.959)	<0.001

DT-ee, diaphragm thickness at the end of expiration; DT-ei, diaphragm thickness at the end of inspiration; DT-edi, diaphragm thickness at the end of deep inspiration; ICC, Intraclass Correlation Coefficient; US physician, Ultrasound physician; CC physician, Critical Care physician.

### Correlation analysis

In this study, we also designed a new indicator named *pleural sliding displacement* (PSD), which was the displacement at the junction of the right lung and liver during the end of expiration and the end of inspiration. Correlation analysis showed a moderate positive correlation between DD-qb and PSD in US physician (*r* = 0.568), CC physician (*r* = 0.470), and total cases (*r* = 0.511), with significant differences (*P* < 0.05).

## Discussion

This study reports the normal reference ranges of various diaphragmatic ultrasound parameters and highlights differences between sexes. Diaphragm ultrasound is a reliable technique with good reproducibility, both intra-and inter-observer. Diverse ultrasound indices can comprehensively assess diaphragm function.

Diaphragmatic displacement (DD) was one of the first indices used by ICU physicians. DD < 10 mm indicates diaphragmatic dysfunction (15), and values between 10 and 14 mm strongly predict extubation failure in mechanical ventilation (16–18). Several factors influence DD, such as elevated intra-abdominal pressure and enhanced respiratory drive. Right-sided DD imaging is generally feasible but may be challenging in cases of obesity, pregnancy, or postoperative patients with tight bandages (19). Insights from lung ultrasound, particularly the “pleural sliding” observed at the interface of the right lung and liver, inspired the development of a new metric: pleural sliding distance (PSD). PSD measures the movement of the lung’s lower boundary during respiration and is conceptually similar to DD. In this study, correlation analysis revealed a moderate positive relationship between DD at quiet breathing (DD-qb) and PSD (*r* = 0.470–0.568, *p* < 0.05). We conclude that DD remains the best index for assessing diaphragmatic contraction and pulmonary ventilation. However, PSD can serve as an alternative when DD is difficult to measure. Additionally, DD measured during maximal inspiration (DD-di) reflects the diaphragm’s maximum contraction capacity. Since DD is highly dependent on subjective inspiratory effort, increased respiratory drive during dyspnea may mask diaphragmatic dysfunction (e.g., a falsely normal DD > 10 mm) (20). Therefore, assessing DD-di is essential to capture the diaphragm’s full contraction reserve. This study found that the DD-qb values for males and females were 16.25 ± 4.05 mm and 14.77 ± 2.61 mm, respectively, during quiet breathing, and 57.04 ± 11.34 mm and 54.86 ± 9.65 mm during maximal inspiration. These findings demonstrate significant diaphragmatic contraction reserve. In dyspneic patients (e.g., during acute pulmonary disease or weaning trials), diaphragmatic atrophy may result in falsely normalized DD values due to enhanced respiratory effort. DD-di, however, offers a more reliable assessment of maximum diaphragmatic contraction. For instance, studies on chronic obstructive pulmonary disease (COPD) have reported higher DD values in patients than in healthy controls (21), potentially due to underlying compensatory diaphragm contraction during hospitalization. Since quiet breathing may not ensure true relaxation, evaluating DD-di is crucial to assess both maximal contraction and reserve capacity.

DT and DTF are also key ultrasound indices. Baseline DT, measured at end-expiration, reflects diaphragm thickness at rest, while inspiratory muscle contraction increases DT proportionally with respiratory effort. A DTF < 20% during quiet breathing is considered indicative of diaphragmatic dysfunction (16). In this study, DT was measured using both quiet and deep breathing modes. DT can be assessed using B-mode or M-mode. While M-mode measurements are simpler, B-mode is more accurate when the pleural line and sampling line are not perpendicular. This study demonstrated good reproducibility for DT measurements across end expiration, end inspiration, and end deep inspiration using B-mode and M-mode (ICC: 0.919–0.960). However, there is no consensus on whether B-mode or M-mode is superior (22). This study suggests that both methods are viable for DT assessment.

TDI, a technique commonly used for myocardial evaluation, has also been applied to the diaphragm (8), however, the normal value has not been confirmed yet. This study showed that normal values for PCV and PRV were established as 2.34 ± 0.30 cm/s and 2.11 ± 0.39 cm/s for males, and 2.30 ± 0.25 cm/s and 2.06 ± 0.34 cm/s for females, respectively. No significant differences were observed (*p* > 0.05). Abnormally high or low TDI values suggest altered diaphragm function, with low TDI indicating reduced contraction velocity and high TDI potentially reflecting increased respiratory drive. For instance, a patient with normal DD but elevated PCV may exhibit pseudo-normalization due to enhanced respiratory effort. This study highlighted lower reproducibility for TDI compared to other indices, possibly due to the low Doppler velocities during quiet breathing. Training and standardization of ultrasound settings and probe positions are recommended for reliable TDI measurements. Jonkman et al. believed that TDI results are very dependent on ultrasound settings and probe position, which may pose certain difficulties for measurements by different observers (23). McCool and Tzelepis believed that the challenges include the influence of thoracoabdominal compliance, adjacent structures like the liver, and potential pleural adhesions, which differentiate diaphragmatic motion from myocardial motion in using TDI (24).

SWE is an ultrasound imaging method based on detecting the propagation of shear waves in tissues, which can measure the stiffness of tissues (9, 25). In recent years, SWE has been increasingly studied in ICU (9). Studies have shown that diaphragm shear modulus values increase with inspiratory pressure (26) and correlate well with transdiaphragmatic pressure (27). This study found that healthy males and females had diaphragm shear modulus values of 15.83 ± 2.01 kPa and 14.92 ± 3.13 kPa at end expiration, and [22.40 (19.90, 25.00)] kPa and [19.90 (17.50, 22.20)] kPa at end deep inspiration, respectively. While males showed higher values at end deep inspiration (*P* < 0.05) and no sex differences were observed at end expiration. SWE demonstrated excellent reproducibility at end expiration and at end deep inspiration in intra-US physician (ICC: 0.947 and 0.908), intra-CC physician (ICC: 0.876 and 0.883), and between US physician and CC physician (ICC: 0.824 and 0.874), respectively. Our results were similar to those of Flatres et al. (9). SWE may help detect disease-induced diaphragm stiffness changes, such as increased stiffness in COPD due to chronic inflammation and oxidative stress (28, 29), or reduced stiffness in diaphragmatic atrophy from ventilator-induced dysfunction. SWE technology will be a good tool for diaphragmatic ultrasound evaluation in the future.

Our research showed that diaphragmatic ultrasound has good reproducibility between ultrasound physicians and critical care physicians, and it is a stable technique. The impact of gender differences on diaphragm ultrasound indicators should be distinguished. Male have higher muscle mass than Female, both in skeletal and respiratory muscles. Our research has some limitations. Firstly, our sample size is not large. Secondly, we did not conduct pulmonary function tests on the subjects, and whether lung capacity and tidal volume are influencing factors of diaphragmatic ultrasound indicators is also a potential issue.

## Conclusion

This study establishes a range of ultrasound-based methods for diaphragm assessment and provides normative reference values. In clinical practice, relying on a single ultrasound index may not suffice for diagnosing or suspecting diaphragmatic dysfunction or atrophy. A combination of techniques offers a more comprehensive evaluation of diaphragm function. Diaphragmatic ultrasound is a practical, bedside, radiation-free monitoring tool. Future large-scale studies are needed to further refine its clinical applications.

## Data Availability

The raw data supporting the conclusions of this article will be made available by the authors, without undue reservation.

## References

[1] Boussuges A Rives S Finance J Brégeon F. Assessment of diaphragmatic function by ultrasonography: current approach and perspectives. World J Clin Cases. (2020) 8:2408–24. doi: 10.12998/wjcc.v8.i12.2408, PMID: 32607319 PMC7322428

[2] Fayssoil A Mansencal N Nguyen LS Orlikowski D Prigent H Bergounioux J. Diaphragm ultrasound in cardiac surgery: state of the art. Medicines. (2022) 9:5. doi: 10.3390/medicines9010005, PMID: 35049938 PMC8779362

[3] Mu H Zhang Q. The application of diaphragm ultrasound in chronic obstructive pulmonary disease: a narrative review. COPD. (2024) 21:2331202. doi: 10.1080/15412555.2024.2331202, PMID: 38634575

[4] Dres M Goligher EC Heunks LMA Brochard LJ. Critical illness-associated diaphragm weakness. Intensive Care Med. (2017) 43:1441–52. doi: 10.1007/s00134-017-4928-4, PMID: 28917004

[5] Marin-Corral J Dot I Boguña M Cecchini L Zapatero A Gracia MP. Structural differences in the diaphragm of patients following controlled vs assisted and spontaneous mechanical ventilation. Intensive Care Med. (2019) 45:488–500. doi: 10.1007/s00134-019-05566-5, PMID: 30790029

[6] Spadaro S Grasso S Mauri T Dalla Corte F Alvisi V Ragazzi R. Can diaphragmatic ultrasonography performed during the T-tube trial predict weaning failure? The role of diaphragmatic rapid shallow breathing index. Crit Care. (2016) 20:305. doi: 10.1186/s13054-016-1479-y, PMID: 27677861 PMC5039882

[7] Poddighe D Van Hollebeke M Choudhary YQ Campos DR Schaeffer MR Verbakel JY. Accuracy of respiratory muscle assessments to predict weaning outcomes: a systematic review and comparative meta-analysis. Crit Care. (2024) 28:70. doi: 10.1186/s13054-024-04823-4, PMID: 38454487 PMC10919035

[8] Soilemezi E Savvidou S Sotiriou P Smyrniotis D Tsagourias M Matamis D. Tissue Doppler imaging of the diaphragm in healthy subjects and critically ill patients. Am J Respir Crit Care Med. (2020) 202:1005–12. doi: 10.1164/rccm.201912-2341OC, PMID: 32614246 PMC7528801

[9] Flatres A Aarab Y Nougaret S Garnier F Larcher R Amalric M. Real-time shear wave ultrasound elastography: a new tool for the evaluation of diaphragm and limb muscle stiffness in critically ill patients. Crit Care. (2020) 24:34. doi: 10.1186/s13054-020-2745-6, PMID: 32014005 PMC6998330

[10] Boon AJ Harper CJ Ghahfarokhi LS Strommen JA Watson JC Sorenson EJ. Two-dimensional ultrasound imaging of the diaphragm: quantitative values in normal subjects. Muscle Nerve. (2013) 47:884–9. doi: 10.1002/mus.23702, PMID: 23625789

[11] Carrillo-Esper R Pérez-Calatayud ÁA Arch-Tirado E Díaz-Carrillo MA Garrido-Aguirre E Tapia-Velazco R. Standardization of sonographic diaphragm thickness evaluations in healthy volunteers. Respir Care. (2016) 61:920–4. doi: 10.4187/respcare.03999, PMID: 27072012

[12] Spiesshoefer J Herkenrath S Henke C Langenbruch L Schneppe M Randerath W. Evaluation of respiratory muscle strength and diaphragm ultrasound: normative values, theoretical considerations, and practical recommendations. Respiration. (2020) 99:369–81. doi: 10.1159/000506016, PMID: 32396905

[13] Boussuges A Rives S Finance J Chaumet G Vallée N Risso JJ. Ultrasound assessment of diaphragm thickness and thickening: reference values and limits of normality when in a seated position. Front Med. (2021) 8:742703. doi: 10.3389/fmed.2021.742703 PMC8579005 34778304

[14] Molina-Hernández N Chicharro JL Becerro-de-Bengoa-Vallejo R Losa-Iglesias ME Rodríguez-Sanz D Vicente-Campos D. Ultrasonographic reliability and repeatability of simultaneous bilateral assessment of diaphragm muscle thickness during normal breathing. Quant Imaging Med Surg. (2023) 13:6656–67. doi: 10.21037/qims-23-329, PMID: 37869345 PMC10585514

[15] Kim WY Suh HJ Hong SB Koh Y Lim CM. Diaphragm dysfunction assessed by ultrasonography: influence on weaning from mechanical ventilation. Crit Care Med. (2011) 39:2627–30. doi: 10.1097/CCM.0b013e3182266408, PMID: 21705883

[16] Santana PV Cardenas LZ Albuquerque ALP Carvalho CRR Caruso P. Diaphragmatic ultrasound: a review of its methodological aspects and clinical uses. J Bras Pneumol. (2020) 46:e20200064. doi: 10.36416/1806-3756/e20200064, PMID: 33237154 PMC7909996

[17] Jiang JR Tsai TH Jerng JS Yu CJ Wu HD Yang PC. Ultrasonographic evaluation of liver/spleen movements and extubation outcome. Chest. (2004) 126:889S–5S. doi: 10.1378/chest.126.1.179, PMID: 15249460

[18] Yoo JW Lee SJ Lee JD Kim HC. Comparison of clinical utility between diaphragm excursion and thickening change using ultrasonography to predict extubation success. Korean J Intern Med. (2018) 33:331–9. doi: 10.3904/kjim.2016.152, PMID: 29050461 PMC5840594

[19] Boussuges A Gole Y Blanc P. Diaphragmatic motion studied by m-mode ultrasonography: methods, reproducibility, and normal values. Chest. (2009) 135:391–400. doi: 10.1378/chest.08-1541, PMID: 19017880

[20] Wang L Muhetaer Y Zhu L Wang Q Wu W. Is it reasonable to predict weaning by measuring diaphragm activity under ultrasound especially its reduction of excursion? Crit Care. (2023) 27:309. doi: 10.1186/s13054-023-04585-5, PMID: 37550661 PMC10408102

[21] Corbellini C Boussuges A Villafañe JH Zocchi L. Diaphragmatic mobility loss in subjects with moderate to very severe COPD may improve after in-patient pulmonary rehabilitation. Respir Care. (2018) 63:1271–80. doi: 10.4187/respcare.06101, PMID: 30065081

[22] Haaksma ME Smit JM Boussuges A Demoule A Dres M Ferrari G. EXpert consensus on diaphragm ultra sonography in the critically ill (EXODUS): a Delphi consensus statement on the measurement of diaphragm ultrasound-derived parameters in a critical care setting. Crit Care. (2022) 26:99. doi: 10.1186/s13054-022-03975-5, PMID: 35395861 PMC8991486

[23] Jonkman AH Wennen M Sklar MC de Korte C Tuinman PR. Tissue Doppler imaging of the diaphragm: a novel approach but too early for clinical implementation? Am J Respir Crit Care Med. (2020) 202:1741–2. doi: 10.1164/rccm.202007-2958LE, PMID: 32961066 PMC7737574

[24] McCool FD Tzelepis GE. Tissue Doppler imaging of the diaphragm: a new kid on the block?[J]. Am J Respir Crit Care Med. (2020) 202:921–2. doi: 10.1164/rccm.202007-2771ED, PMID: 32749867 PMC7528794

[25] Hug F Tucker K Gennisson JL Tanter M Nordez A. Elastography for muscle biomechanics: toward the estimation of individual muscle force. Exerc Sport Sci Rev. (2015) 43:125–33. doi: 10.1249/JES.0000000000000049 25906424

[26] Chino K Ohya T Katayama K Suzuki Y. Diaphragmatic shear modulus at various submaximal inspiration mouth pressure levels. Respir Physiol Neurobiol. (2018) 252-253:52–7. doi: 10.1016/j.resp.2018.03.009, PMID: 29567109

[27] Fossé Q Poulard T Niérat MC Virolle S Morawiec E Hogrel JY. Ultrasound shear wave elastography for assessing diaphragm function in mechanically ventilated patients: a breath-by-breath analysis. Crit Care. (2020) 24:669. doi: 10.1186/s13054-020-03338-y, PMID: 33246478 PMC7695240

[28] Xu JH Wu ZZ Tao FY Zhu ST Chen SP Cai C. Ultrasound shear wave elastography for evaluation of diaphragm stiffness in patients with stable COPD: a pilot trial. J Ultrasound Med. (2021) 40:2655–63. doi: 10.1002/jum.15655, PMID: 33615538

[29] Chen Y Li J Dong B Zhu Z Lyu G. Two-dimensional shear wave elastography: a new tool for evaluating respiratory muscle stiffness in chronic obstructive pulmonary disease patients. BMC Pulm Med. (2022) 22:441. doi: 10.1186/s12890-022-02231-4, PMID: 36424581 PMC9686016

